# Effects of Drip Irrigations with Different Irrigation Intervals and Levels on Nutritional Traits of Paddy Cultivars

**DOI:** 10.3390/foods14030528

**Published:** 2025-02-06

**Authors:** Beyza Ciftci, Yusuf Murat Kardes, Ihsan Serkan Varol, Ismail Tas, Sevim Akcura, Yalcin Coskun, Kevser Karaman, Zeki Gokalp, Mevlut Akcura, Mahmut Kaplan

**Affiliations:** 1 Department of Field Crops, Faculty of Agriculture, University of Erciyes, Kayseri 38000, Turkey; beyzacftc.58@gmail.com (B.C.); mahmutkaplan5@hotmail.com (M.K.); 2 Department of Field Crops, Faculty of Agriculture, Bilecik Seyh Edebali University, Bilecik 11000, Turkey; 3 Department of Biosystems Engineering, Faculty of Agriculture, University of Erciyes, Kayseri 38000, Turkey; ihsanserkanvarol@gmail.com (I.S.V.); zgokalp@erciyes.edu.tr (Z.G.); 4 Department of Agricultural Structures and Irrigation, Faculty of Agriculture, Canakkale Onsekiz Mart University, Canakkale 17000, Turkey; 5 Department of Field Crops, Institute of Natural and Applied Sciences, Canakkale Onsekiz Mart University, Canakkale 17020, Turkey; sevimakcura@yahoo.com; 6 Lapseki Vocational College, Canakkale Onsekiz Mart University, Canakkale 17000, Turkey; ycoskun@comu.edu.tr; 7 Department of Agricultural Biotechnology, Faculty of Agriculture, University of Erciyes, Kayseri 13000, Turkey; kevserkaraman@erciyes.edu.tr; 8 Department of Field Crops, Faculty of Agriculture, Canakkale Onsekiz Mart University, Canakkale 17100, Turkey; makcura@comu.edu.tr

**Keywords:** paddy, irrigation interval, irrigation level, cultivar, nutritional traits

## Abstract

Rice serves as the primary food source for the majority of the world’s population. In terms of irrigation water, the highest volume of irrigation water is utilized in paddy irrigation. Excessive water use causes both waste of limited water resources and various environmental problems. The drip irrigation method with high water use efficiency will reduce both the need for irrigation water and the environmental footprint of paddy production. This study was conducted to investigate the effects of two different irrigation intervals (2 and 4 days) and four irrigation levels (150%, 125%, 100%, and 75% of evaporation from a Class-A pan) on the nutritional traits of three different paddy cultivars (Ronaldo, Baldo, and Osmancık). Increasing irrigation intervals and decreasing irrigation levels reduced the nutritional properties (protein, oil, starch) of the rice grains. In addition, increasing irrigation levels also increased the phytic acid and dietary fiber contents. The highest protein (7.14%) and total starch (87.10%) contents were obtained from the 150% irrigation treatments. The highest amylose content (20.74%) was obtained from the 75% irrigation treatment. In general, it was found that irrigation levels should be applied at 125% and 150% to increase the mineral content of rice grains. Although water deficits decreased the nutritional properties of the paddy cultivars, drip irrigation at an appropriate level did not have any negative effects on nutritional traits.

## 1. Introduction

On-going global warming and climate change pose serious risks to crop production [1]. The world population is also continuously increasing. Therefore, maximum yields with sufficient quality should be achieved from limited sources to meet the food demands of the rapidly growing world population [2,3]. While doing so, agricultural practices should not pose any risks to environmental, soil, and water resources [4].

Rice (*Oryza sativa* L.) is an essential source of food for more than half of the world’s population [5,6] and is among the top three cultivated and produced plants of the world [7]. Since millions of poor families depend heavily on rice for their diets, it makes a significant contribution to the daily calorie intake of poor people [8,9]. It is a good source of carbohydrates, with more than 80% starch in rice grains. Besides its high protein content (7–8%), its protein quality, as indicated by its protein digestibility index and amino acid composition, is also better than that of other cereal grains [10], and this makes it preferable for the food and feed industries. For rice plants to grow and develop, water is necessary. Continuous flooding operations are used to generate more than 75% of the rice consumed worldwide [11,12].

Starch is one of the most important components of rice, constituting approximately 80% of the grain. While waxy varieties contain almost no amylose, non-waxy varieties can contain up to 35% amylose. The properties of starch vary depending on its amylose, amylopectin, and mineral contents. The amylose content influences characteristics such as pasting, gelatinization, and retrogradation. Previous studies have shown that the protein, amylose, and starch characteristics of warm-climate cereals such as corn and sorghum are significantly affected by irrigation and genotypes [13,14,15].

Irrigation is a must in paddy farming [16]. Ever-depleting water resources, production costs, and environmental factors have led to the need to produce rice with less irrigation water. Increasing grain yield and maintaining grain quality while reducing water use has become a major challenge for the global rice industry [17]. Recently, studies have been conducted on this issue in several parts of the world [18,19,20,21]. These studies mostly focused on yield and water use efficiency, but there are very few studies on the effect of irrigation intervals and irrigation water quantities on the nutritional properties of paddy cultivars.

Irrigation water supply and management are critical issues and limiting factors in paddy farming [22]. Rice paddies, which provide a staple food for more than half of the world’s population, consume a large amount of water [23]. Mallaredy et al. [24] reported that 34–45% of global irrigation water consumption is used in paddy farming, making rice one of the most water-intensive crops in the world. The depletion of water resources for rice cultivation can lead to soil degradation and a decrease in agricultural productivity. The excessive use of water resources can also lead to increased salinity and waterlogging, further reducing agricultural productivity [25]. In Turkey, the water consumption of paddy plants is estimated to be between 810 and 1625 mm depending on climate conditions. It was stated that 1000–1200 L of water is sufficient for 1 kg of rice production, but such a quantity sometimes reaches 4000–5000 L in practice [26]. Despite increasing populations, water resources are gradually decreasing, and paddy farming corresponds to 35% of the total irrigation water used worldwide [27]. There is a strong relationship between soil moisture content and the availability of plant nutrients. Sufficient soil moisture has been reported to increase the protein, starch, and oil contents of rice grains [28,29]. Therefore, this study was conducted to reduce the amount of irrigation water and the cost of water to determine the response of locally grown varieties to irrigation and to determine the effect of different irrigations on the nutritional traits of paddy cultivars.

## 2. Materials and Methods

### 2.1. Field Experiments

The experiments were conducted in Çanakkale Province in Turkey, located between 25°40′–27°30′ east longitudes and 39°27′–40°45′ north latitudes. A randomized block split-plot experimental design with three replications was adopted. In the first week of May 2017, 500 seeds per m^2^ were manually sown in rows opened with a marker. In the experiments, the Ronaldo, Baldo, and Osmancık varieties, which are widely cultivated and commonly used in the region, were utilized. Before sowing, DAP (diammonium phosphate) fertilizer was applied in order to have 100 kg P_2_O_5_ and 39 kg N per hectare. During the tillering and panicle formation period, a total of 100 kg ha^−1^ N (50 kg ha^−1^ in each period) was applied with the use of 21% N-containing ammonium sulphate fertilizer. Chemical control was performed against broad- and narrow-leaved weeds. Harvest was practiced in the last week of October with a plot thresher.

### 2.2. Climate Characteristics

Although Çanakkale’s climate has a transitional nature due to its geographical location, it largely shows the characteristics of the Mediterranean climate. Some climate parameters measured in the study year are presented in Table 1. The hottest months were determined to be July and August. There was no precipitation in these months. July was determined to be the month with the highest sunshine duration and the lowest relative humidity (57.9%).

### 2.3. Design and Practice of Irrigations

The drip irrigation method was used in the present experiments. In the first irrigation, sufficient irrigation water was applied to bring the soil available moisture to field capacity, and then equal amounts of irrigation water were applied to all treatments for a period of four weeks until the seedling root system developed. After the plants achieved sufficient growth, irrigation treatments were initiated. The amount of irrigation water to be applied to each treatment was determined with the use of a Class-A evaporation pan placed on the experimental fields. Measured cumulative open water surface evaporation values were applied at 2- and 4-day intervals. As the irrigation levels, 150%, 125%, 100%, and 75% cumulative evaporation values were applied. The amount of irrigation water to be applied was calculated as suggested by Kanber et al. [30]:

(1) $$I=E_{pan}\times K_{p}$$

where

*I*: amount of irrigation water to be applied (mm);

*E_Pan_*: cumulative evaporation from Class-A pan (mm);

*K_p_*: pan coefficient.

As recommended by Eylen et al. [31], the pressure–dripper flow rate–time relationship was used for the assessment of the amount of irrigation water applied. The following equation was used for this purpose:

(2) $$T=\frac{I\times A}{q\times n}$$

where

*T*: irrigation duration (h);

*I*: amount of irrigation water to be applied (mm);

*A*: plot area (m^2^);

*q*: dripper flow rate at operational pressure (L/h);

*n*: number of drippers in each plot.

The total irrigation water applied during the production period and actual evapotranspiration values are shown in Table 2.

### 2.4. Nutritional Traits

For biochemical analyses, harvested paddies were dehulled and cleaned. Bare grains were then turned into a homogeneous powder using a grinder and stored at −18 °C until analysis.

#### 2.4.1. Crude Protein Content

Protein quantification was performed using the Kjeldahl method [32]. About 1 g of sample was acid-digested in 25 mL of concentrated H_2_SO_4_. Following the digestion process, the distillation stage was started. The burning process was completed at 380 °C by gradually increasing the temperature. The distillate was titrated with 0.2 N HCl solution, and the amount of HCl used in titration was recorded to calculate the total nitrogen and protein contents.

#### 2.4.2. Phytic Acid

The phytic acid content of the paddy samples was measured with the use of high-performance liquid chromatography (HPLC). Before HPLC analysis, all paddy samples were dried and milled. Then, 40 mg of each sample was extracted with 1.6 mL of 0.4 mol/L HCl overnight and centrifuged at 5000× *g* for 10 min. The supernatants were filtered through a 0.45 µm filter, and readings were performed via HPLC (Shimadzu, Kyoto, Japan) equipped with a PDA detector. Samples were loaded onto a 4.6 × 250 mm SunFire C18 column (Waters, Milford, MA, USA). Elution was performed at a flow rate of 1 mL/min (40 °C) using a mobile phase consisting of acetonitrile and distilled water (50%:50% *w*/*w*) to be adjusted to pH 4.5 with formic acid. Phytic acid was detected at a wavelength of 195 nm [33,34].

#### 2.4.3. Resistant, Non-Resistant, and Total Starch

A Megazyme Resistant Starch Analysis Kit, developed based on the AOAC [32] Method and AACC [35] Method, was used to determine the resistant starch content. In this method, from rice flour samples incubated for 16 h at 37 °C in the presence of α-amylase and amyloglucosidase, non-resistant starch is dissolved in the first stage and broken down into glucose. Resistant starch is obtained from the sediment part after the centrifugation process, and after stages such as washing with ethanol and dissolving in potassium hydroxide, it is hydrolyzed into glucose by the amyloglycosidase enzyme. Resistant starch and total starch values were obtained separately by spectrophotometric determination of glucose.

#### 2.4.4. Amylose/Amylopectin

A Megazyme Amylose/Amylopectin Analysis Kit was used to determine the amylose and amylopectin fractions of starch. Starch samples were completely dispersed in a boiling water bath (98 °C) by adding dimethyl sulfoxide, and starch was precipitated by adding ethanol. Oils were removed by recycling the precipitate. The starch precipitate was dissolved in acetate, conA was added, and amylopectin was removed by centrifugation. Afterwards, amylose and total starch were hydrolyzed into D-glucose, and the amylose value was determined spectrophotometrically by the addition of glucose oxidase peroxidase.

### 2.5. Mineral Composition

For mineral analysis (P, K, Ca, Mg, Na, Fe, Mn, Zn, Cu, and B), about 0.5 g of sample was supplemented with 10 mL of a nitric + perchloric acid mixture, and the resultant mixture was subjected to wet digestion until approximately 1 mL of sample remained. Resultant solutions were diluted with distilled water, and readings were performed using an ICP OES spectrophotometer (inductively coupled plasma spectrophotometer) (Perkin-Elmer, Optima 4300 DV, ICP/OES, Shelton, CT 06484-4794, USA) [36].

### 2.6. Statistical Analyses

Biochemical data were analyzed using JMP Pro 17 (SAS Institute, Cary, NC, USA) [37] software in accordance with a randomized block split-plot experimental design. Experimental data were subjected to variance analysis to determine the effects of the processing variables on the studied parameters. The LSD test was used for multiple comparisons.

## 3. Results

### 3.1. Amylose and Amylopectin

The amylose and amylopectin contents of all paddy cultivars exhibited significant variations with the irrigation treatments (Supplementary Materials Tables S1 and S2).

In general, 2- and 4-day irrigation intervals yielded similar outcomes. In terms of irrigation levels, amylopectin contents showed a positive and amylose contents showed a negative linear quadratic relationship. In all cultivars, the lowest amylopectin and highest amylose contents were obtained from the 75% irrigation treatments for both irrigation intervals, while the highest amylopectin and lowest amylose contents were obtained from the 150% irrigation treatments (Supplementary Materials Tables S1 and S2).

**Figure 1:**
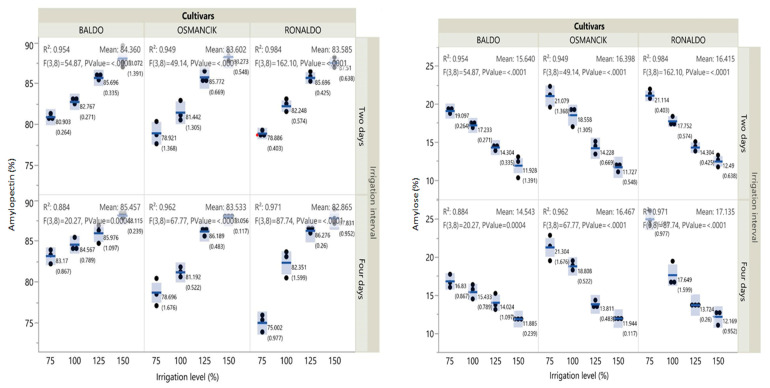

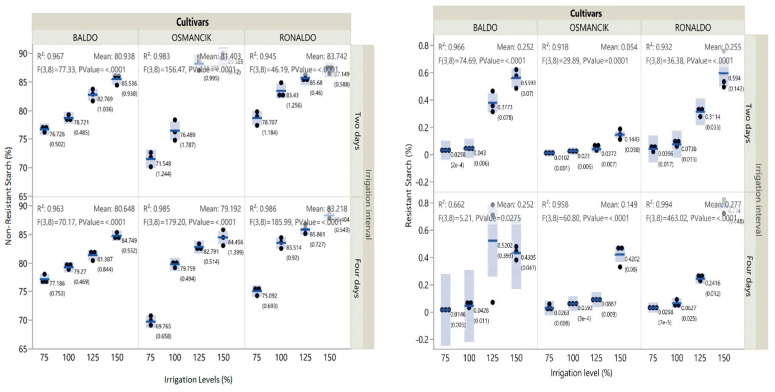
Effects of irrigation intervals and levels on amylopectin, amylose, and resistant and non-resistant starch contents of paddy cultivars (F: F-test; numbers in parentheses: standard error; lines between the dots: median; number: mean; the bars represent increases and decreases in values).

When all factors included in the research and their interactions were evaluated together, it was observed that amylopectin contents increased and amylose contents decreased with increasing irrigation levels (Figure 1, Supplementary Materials Tables S1 and S2).

### 3.2. Resistant Starch (RS), Non-Resistant Starch (NRS), and Total Starch (TS)

The differences in the RS, NRS, and TS contents of the studied paddy cultivars were found to be significant (*p* < 0.01). The Ronaldo cultivar yielded the greatest RS, NRS, and TS concentrations at both watering intervals (Supplementary Materials Tables S1 and S2).

**Figure 2:**
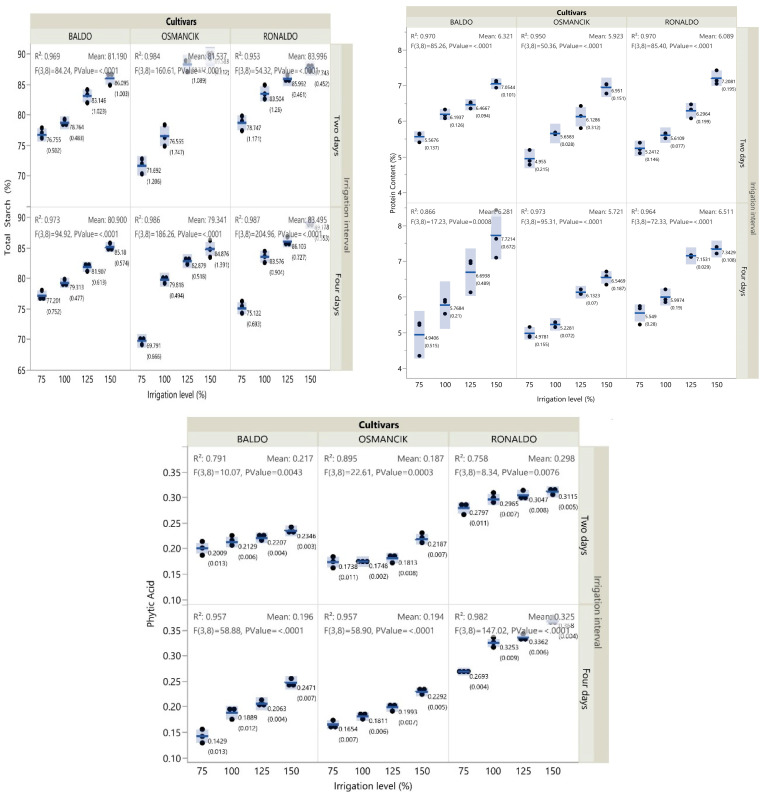
Change in total starch, protein, and phytic acid contents of paddy cultivars with irrigation treatments (F: F-test; numbers in parentheses: standard error; lines between the dots: median; number: mean).

The lowest RS and NRS contents were obtained from the Osmancık cultivar. The total starch contents of all cultivars increased linearly with increasing irrigation levels. The change in the NRS ratio of the paddy cultivars with irrigation levels was found to be significant (*p* < 0.01). The lowest non-resistant starch contents were obtained from the 75% irrigation treatments and the highest from the 150% irrigation treatments (Figure 1, Supplementary Materials Tables S1 and S2). The resistant starch contents tended to increase in all cultivars with increasing irrigation levels. However, the coefficient of determination for the change in the resistant starch content of the Baldo cultivar at the 4-day irrigation interval was lower. In all cultivars, the lowest total starch content was obtained from the 75% irrigation treatments and the highest from the 150% irrigation treatments (Figure 2, Supplementary Materials Tables S1 and S2).

### 3.3. Protein Content

In terms of the protein ratio, cultivars, irrigation levels, irrigation intervals, and the double and triple interactions of these factors were all found to be significant. In all cultivars, the protein contents increased with increasing irrigation levels at both irrigation intervals (Figure 2). The highest protein contents were obtained from the 150% irrigation treatments. The increase in the protein ratios was close to linear. The lowest protein contents were obtained from the 75% and 100% irrigation treatments of the Osmancık cultivar at the 4-day irrigation interval (Supplementary Materials Tables S1 and S2).

### 3.4. Phytic Acid

In terms of the phytic acid contents, cultivars, irrigation levels, and irrigation intervals were found to be significant (*p* < 0.01). The lowest phytic acid content was obtained from the Osmancık cultivar. At the 4-day irrigation interval, the phytic acid content of all cultivars increased linearly with increasing irrigation levels. However, such changes in the phytic acid contents of the cultivars were lower at the 2-day irrigation interval (Figure 2, Supplementary Materials Tables S1 and S2).

### 3.5. Mineral Composition

Except for calcium, all variation sources and double and triple interactions of the factors were found to be significant for the minerals (B, Cu, Fe, K, Mg, Mn, Na, P, S, and Zn) (*p* < 0.01). In terms of the Ca content, cultivars and irrigation levels were found to be significant, irrigation intervals and irrigation level x irrigation interval interactions were found to be insignificant, and the other interactions were found to be significant (*p* < 0.01) (Supplementary Materials Tables S3 and S4).

**Figure 3:**
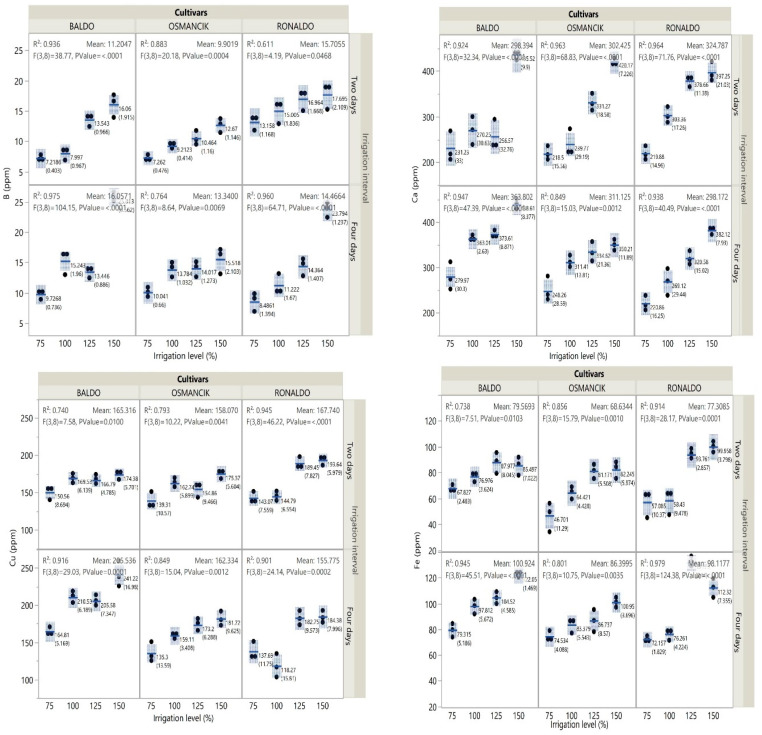
Change in B, Ca, Cu, and Fe contents of paddy cultivars with irrigation treatments.

Among the cultivars, the highest boron content was obtained from the Ronaldo cultivar. In terms of irrigation intervals, the highest boron contents were obtained from the Baldo and Osmancık cultivars at the 4-day irrigation interval and from the Ronaldo cultivar at the 2-day irrigation interval. Generally, in all cultivars, the lowest boron content was obtained from the 75% irrigation treatments and the highest from the 150% irrigation treatments. Among the cultivars, the highest calcium content was obtained from the Baldo cultivar and the lowest from the Ronaldo cultivar. Generally, in all cultivars, the lowest calcium content was obtained from the 75% irrigation treatments and the highest from the 150% irrigation treatments (Figure 3, Supplementary Materials Tables S3 and S4).

In terms of the cultivars, the highest copper content was obtained from the Baldo cultivar and the lowest from the Ronaldo cultivar. The copper contents of the Baldo and Osmancık cultivars detected at the 4-day irrigation interval were higher than those detected at the 2-day irrigation interval (Figure 3, Supplementary Materials Tables S3 and S4). While the Osmancık cultivar had the lowest iron content, the Baldo cultivar had the highest iron content. In all three cultivars, greater iron contents were seen at the 4-day irrigation interval than at the 2-day irrigation interval. When evaluated over all sources of variation, iron contents tended to increase with increasing irrigation levels. Except for the 4-day irrigation interval of the Ronaldo cultivar, in the other cultivar × irrigation level interactions, the highest iron content was obtained from the 125% irrigation treatments and the lowest from the 75% irrigation treatments (Figure 3, Supplementary Materials Tables S3 and S4).

**Figure 4:**
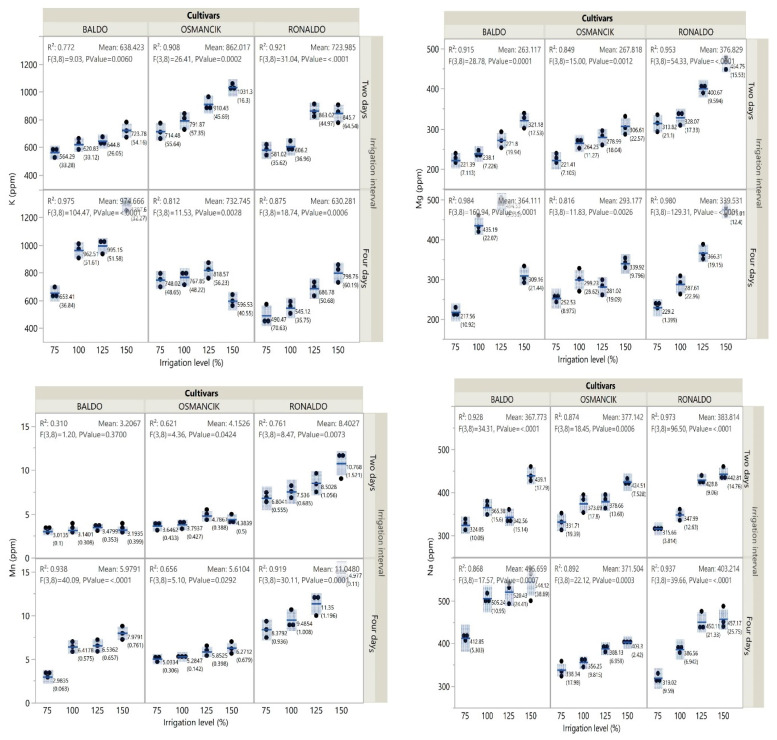
Change in K, Mg, Mn, and Na contents of paddy cultivars with irrigation treatments.

The Ronaldo cultivar had a lower potassium content than the other two cultivars. In general, high potassium levels were determined in the Osmancık cultivar at the 2-day irrigation interval, while high potassium levels were determined in the Baldo cultivar at the 4-day irrigation interval (Figure 4, Supplementary Materials Tables S3 and S4).

Among the cultivars, the highest magnesium content was detected in the Ronaldo cultivar. In terms of irrigation intervals, greater magnesium contents were obtained at the 4-day irrigation interval than at the 2-day irrigation interval, except for the Ronaldo cultivar (Figure 4, Supplementary Materials Tables S3 and S4). The highest manganese content was obtained from the Ronaldo cultivar. Generally, the manganese contents determined at the 4-day irrigation interval were higher than those at the 2-day irrigation interval (Figure 4, Supplementary Materials Tables S3 and S4). The Baldo cultivar had the highest sodium content. The sodium content of the Osmancık and Ronaldo cultivars was affected by irrigation intervals. The Baldo cultivar had higher sodium contents at the 4-day irrigation interval than at the 2-day irrigation interval. The changes in the sodium content of the Osmancık and Ronaldo cultivars at both irrigation intervals were similar. The sodium contents of these cultivars increased with increasing irrigation levels (Figure 4, Supplementary Materials Tables S3 and S4).

**Figure 5:**
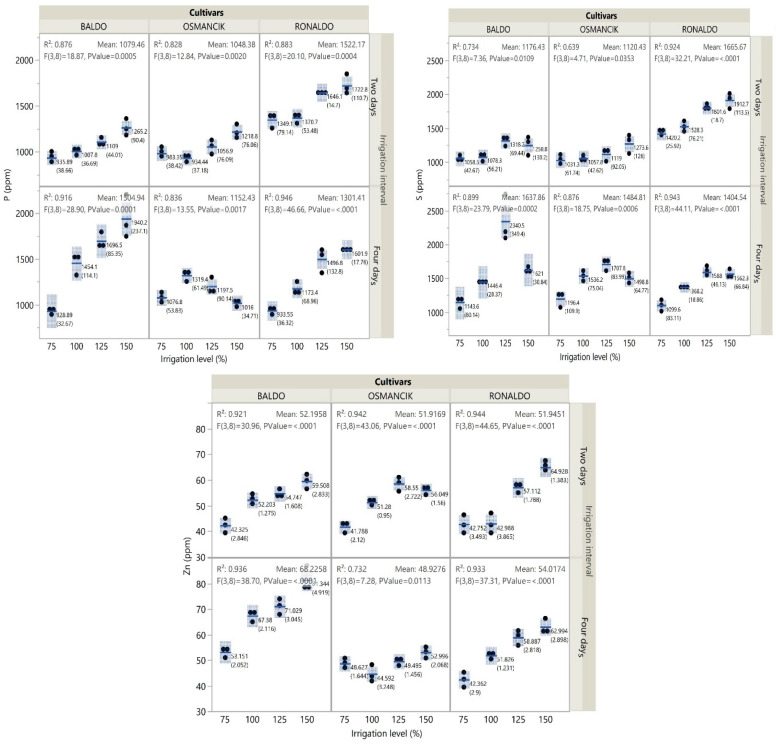
Change in P, S, and Zn content of paddy cultivars with irrigation treatments.

Among the cultivars, the highest phosphorus content was obtained from the Ronaldo cultivar at the 2-day irrigation interval. At the 2-day irrigation interval, phosphorus contents increased in all cultivars with increasing irrigation levels. At the 4-day irrigation interval, an increase was encountered in the phosphorus content of the Baldo and Ronaldo cultivars with increasing irrigation levels, but an increase and then a decrease were observed in the Osmancık cultivar (Figure 5, Supplementary Materials Tables S3 and S4). Among the cultivars, the Ronaldo cultivar had the highest sulfur content at the 2-day irrigation interval. The sulfur contents of the Baldo and Osmancık cultivars were greater at the 4-day irrigation interval than at the 2-day irrigation interval. At the 2-day irrigation interval, the sulfur contents of the paddy cultivars increased with increasing irrigation levels. At the 4-day irrigation interval, the sulfur contents of the cultivars initially increased and then decreased with increasing irrigation levels (Figure 5, Supplementary Materials Tables S3 and S4).

The Baldo cultivar had the highest zinc content. While the zinc content of the Osmancık cultivar was lower at the 4-day irrigation interval than at the 2-day irrigation interval, the opposite was seen in the other two cultivars (Figure 5). The zinc content of the Baldo and Ronaldo cultivars increased with increasing irrigation levels at both irrigation intervals. On the other hand, in the Osmancık cultivar, the zinc content initially increased and then decreased with increasing irrigation levels at the 2-day irrigation interval, while it initially decreased and then increased at the 4-day irrigation interval (Supplementary Materials Tables S3 and S4).

## 4. Discussion

The effects of irrigation intervals and irrigation levels on the biochemical properties of paddy grains were emphasized in this study. It was reported that water stress and excessive irrigation (excessively wet conditions) significantly affected paddy yield and grain quality [38]. However, the responses of cultivars to water deficits or excessive irrigation vary greatly [39]. Therefore, optimization of irrigation water quantities for paddy cultivars is essential for the sustainability of paddy production.

### 4.1. Nutrients

Previous studies reported protein contents of rice flour between 6.93 and 8.33%, oil contents between 0.87 and 1.44%, and amylose contents between 18.1 and 31.6% [40,41]. The present findings agree with the results of previous researchers.

Changes in the transport of assimilates under water stress conditions and resultant changes in metabolic and enzyme activities ultimately alter the chemical composition of paddy grains [28]. In plants under stress, the amount of carbohydrates decreases due to the closure of the stomata in the leaves, and carbohydrate and protein metabolites such as proline and glycine accumulate in the leaves [42]. These nutrients accumulated in the leaves cannot be transported to the grain due to water deficits; thus, there is a decrease in the protein content of the grains. Kırnak et al. [43] reported that water stress reduced both the protein and oil contents of soybean seeds. It was also reported that water stress increased the protein and oil contents of onion [44] and sunflower [45]. Such differences were mainly attributed to differences in environmental conditions, agricultural practices, and genotypes [46]. In this study, protein contents increased with increasing irrigation levels. Although protein is not the main component of starch granules, complexes of proteins and lipids can easily form and bind to the surface or enter into starch granules, thus affecting the properties of starch granules [47]. The total soluble sugars of rice grains can be converted into sucrose and starch, and their quantities indicate starch accumulation [48]. Moderate drought stress accelerates the transport of stored photosynthate during the grain-fill period. These findings revealed that mild water stress promoted the conversion of total soluble sugars and sucrose into starch, increasing the starch content of rice grains by regulating key enzymes involved in starch synthesis [49]. Our study found that the highest starch content was obtained from the 150% irrigation level applied at 2-day intervals. It is thought that the reason for this may be that more frequent irrigation prevented the grains from experiencing stress and enhanced their performance. Under aerobic conditions, reducing sugars increase significantly while starch and non-reducing sugars decrease. Soluble sugars allow plants to maximize carbohydrate storage but facilitate vitrification under water stress, thus preventing damage to cells [50]. In grains, decreases in non-reducing sugars and starch under stress conditions also indicate greater utilization of carbohydrates to overcome stress. Non-reducing sugars in the form of sucrose may be critical sugars providing water stress tolerance under aerobic conditions in both leaves and grains. Such a case is also supported by the activity changes of sucrose synthase/sucrose phosphate synthase and invertases, which are key enzymes controlling sucrose metabolism [51].

The amylose content is the most widely used indicator of rice grain quality, as well as the most frequently used quantitative property of starch. The solubility of starch is dominated by the amylose content, but amylopectin mainly contributes to the swelling power of starch [52]. Sar et al. [53] indicated that the amylose content of rice grains is controlled by genetic factors, while [29] reported that agronomic practices such as irrigation and fertilization, as well as genetic structure, affect the biochemical properties of rice grains. In [54], where water deficits were applied after flowering, it was reported that the amylose content decreased in a variety with a short vegetation period, but a variety with a long vegetation period was not affected by the water deficits. However, a study conducted on rice varieties reported that drought stress suppressed the Wx gene and significantly reduced the amylose content [55]. As is the case with drought stress, excessive irrigation (such as flood) also reduces grain yield and protein content and increases amylose and ash contents [56]. In this study, it was determined that the amylose content of the rice varieties was not affected by the irrigation interval and level. The amylose structure in the rice varieties may be genetically more stable, and environmental factors may have had less influence.

Phytic acid, with an ability to bind proteins and some micronutrients such as iron and zinc, is known as an antinutritional factor and results in a significant decrease in the bioavailability of these nutrients [57]. Fernando [58,59] stated that the grain phytate concentration increased with increasing irrigation levels, while [29] reported that increasing water levels reduced phytic acid contents.

Plant oil contents tend to decrease under dry conditions. Water deficiency facilitates lipolytic and peroxidative activities, as well as inhibition of lipid biosynthesis [60], and these processes are associated with a decrease in the membrane lipid content. However, such a case largely depends on plant species or varieties and environmental conditions [61].

### 4.2. Nutrient Elements

Plant mineral contents are largely dependent on soil characteristics, genotypes, growing conditions, and soil minerals. Additionally, the duration of water stress during plant development plays an important role in the mineral content [62]. Drought plays a direct role in the mineral uptake of plants. There is a positive relationship between soil moisture and the nutrient uptake of roots [63]. As related to reduced transpiration rates, drought can suppress the acropetal translocation of nutrients and impair active transport and membrane permeability [64]. A poorly developed root system reduces the uptake of several nutrients from the soil. In this study, deficit irrigations decreased the nutrient uptake of the plants. The higher mineral uptake rates of drought-resistant cultivars compared with others [65] explain the reason for the difference in the mineral contents of the cultivars.

High K and Ca concentrations were encountered in deficit irrigation treatments [66]. There are considerable differences in studies examining the effect of water stress on Ca and Mg concentrations. While some studies report a general increase with drought [67], others report a decrease [68]. In the present study, decreases were seen in Ca and Mg concentrations with decreasing irrigation levels. Drought stress causes decreases in total P accumulation in plants and has detrimental effects on plant development [69].

Water deficits affect enzyme activity and protein synthesis and reduce photosynthetic pigments (Mg, Mn, Fe, and Zn) in plants [70]. Plant Mg contents decrease significantly with increasing drought levels [71]. A Mg-induced decrease then negatively affects photosynthetic activity and grain formation [72]. Drought causes Mg deficiency and then decreases leaf chlorophyll content, stomatal density, and photosynthetic activity [73].

Ibrahim et al. [74] reported that the nitrogen, phosphorus, and potassium contents of paddy varieties were influenced by irrigation intervals, varieties, and their interactions, and they stated that nitrogen, phosphorus, and potassium decreased as the irrigation interval increased. Verma and Srivastav [75] determined the Zn, Na, Mg, Fe, Cu, and Ca contents of their paddy varieties to be 9.30–17.00 mg/kg, 41.44–68.85 mg/kg, 83.50–182.45 mg/kg, 0.80–31.50 mg/kg, 4.10–15.95 mg/kg, and 62.95–98.75 mg/kg, respectively, and reported differences among the varieties. In rice varieties grown using traditional irrigation and fertilization methods, the iron content in bran was determined to be between 83.73 and 150.47, while the zinc content ranged from 37.51 to 55.89 µg g^−1^ and Mg ranged from 6401 to 11,544 µg g^−1^ [76]. In a rice breeding study using a traditional irrigation method, Zhang et al. [77] reported that the nitrogen (N) content in the glume, bran, and endosperm accounted for 3.8–4.9%, 19.7–21.9%, and 74.3–75.5% of the content in the grains, respectively, while the phosphorus (P) content in the glume, bran, and endosperm accounted for 0.5–0.8%, 98.9–99.1%, and 0.3–0.6% of the content in the grains, respectively.

## 5. Conclusions

The present findings revealed that water stress negatively affected the nutritional properties of all cultivars. With increasing irrigation intervals and decreasing irrigation levels, a decrease was seen in the nutritional properties of the rice grains, such as protein and starch. In addition, increasing irrigation levels also increased the phytic acid content, which reduced the nutrient contents. Currently, increasing water deficits and food demands are making cultivars more tolerant to drought stress, which holds promise for sustainable paddy farming. Further research is needed on the yield and nutritional properties of different rice cultivars under various irrigation levels. Since drip irrigation at appropriate irrigation levels does not cause any loss of nutritional properties in rice grains, paddy irrigation can be carried out with drip irrigation without excessive water use.

## Figures and Tables

**Table 1:** Climate data for the study area.

Months	Tort (°C)	Tmax (°C)	Tmin (°C)	Averge Sunshine Duration (hours)	Average Precipitation (mm)	Average Wind Speed (m/s)	Average Relative Humidity (%)
1	7.2	10.9	3.2	3.6	110.2	4.8	73.6
2	10.9	14.7	7.7	4.2	88.4	5.0	72.2
3	11.2	14.8	7.5	5.3	53.6	4.6	70.8
4	15.8	20.8	11.2	8.4	15.0	4.1	69.0
5	18.3	22.5	14.0	8.5	26.8	3.6	67.3
6	24.5	29.9	19.6	11.2	39.9	3.5	62.2
7	27.0	32.5	22.0	12.2	0.0	4.1	57.9
8	27.0	32.5	22.5	10.8	0.0	4.3	58.2
9	22.5	27.6	17.7	8.5	1.8	4.0	62.6
10	17.1	21.3	12.7	5.8	8.6	4.0	68.4
11	12.5	16.1	8.2	3.1	210.3	4.2	72.4
12	4.9	8.9	1.2	0.22	16.3	4.7	73.9
Aver./Year	16.6	21.0	12.3	6.8	570.9	4.2	67.4

**Table 2:** Total irrigation water amounts applied and actual evapotranspiration values of the trial subjects.

Day	Treatments	Cultuvars	Irrigation Water (mm)	ETa (mm)
2	75	Baldo	513	565
	100	Baldo	615	659
	125	Baldo	718	753
	150	Baldo	820	845
	75	Osmancık	513	565
	100	Osmancık	615	659
	125	Osmancık	718	753
	150	Osmancık	820	845
	75	Ronaldo	513	565
	100	Ronaldo	615	659
	125	Ronaldo	718	753
	150	Ronaldo	820	845
4	75	Baldo	513	580
	100	Baldo	615	673
	125	Baldo	718	763
	150	Baldo	820	855
	75	Osmancık	513	580
	100	Osmancık	615	673
	125	Osmancık	718	763
	150	Osmancık	820	855
	75	Ronaldo	513	580
	100	Ronaldo	615	673
	125	Ronaldo	718	763
	150	Ronaldo	820	855

## Data Availability

The original contributions presented in this study are included in the article/Supplementary Material. Further inquiries can be directed to the corresponding author.

## Supplementary Materials

The following supporting information can be downloaded at: https://www.mdpi.com/article/10.3390/foods14030528/s1, Table S1: The effects of different ırrigation levels and ıntervals on the biochemical properties of rice varieties; Table S2: The variations in biochemical properties of rice varieties with different ırrigation levels; Table S3: The effects of different ırrigation levels and ıntervals on the mineral content of rice varieties; Table S4: The variations in mineral content of rice varieties with different ırrigation levels.
