# Supplementary material for: Effects of Drip Irrigations with Different Irrigation Intervals and Levels on Nutritional Traits of Paddy Cultivars
Source: Foods. 2025 Feb 6;14(3):528. doi: 10.3390/foods14030528 (PMC11817345; doi:10.3390/foods14030528)
Supplement: Supplementary file 1 [file foods-14-00528-s001.zip › foods-3421993-supplementary.pdf]

Table S1. The effects of different ırrigation levels and ıntervals on the biochemical properties of rice varieties

Amylose (%)

Cultivars/IL	2 Days					4 Days					
	75%	100%	125%	150%	Mean	75%	100%	125%	150%	Mean	Mean
**Baldo**	19.10	17.23	14.30	11.93	**15.64^{c}**	16.83	15.43	14.02	11.88	**14.54^{d}**	***15.09 B***
**Osmancık**	21.08	18.56	14.23	11.73	**16.40^{b}**	21.30	18.81	13.81	11.94	**16.47^{b}**	***16.43 A***
**Ronaldo**	21.11	17.75	14.30	12.49	**16.42^{b}**	25.00	17.65	13.72	12.17	**17.13^{a}**	***16.78 A***
**Mean**	**20.43^{ns}**	**17.85^{ns}**	**14.28^{ns}**	**12.05^{ns}**	***16.15^{ns}***	**21.04^{ns}**	**17.30^{ns}**	**13.85^{ns}**	**12.00^{ns}**	***16.05^{ns}***	

Amylopectin (%)

Cultivars/IL	2 Days					4 Days					
	75%	100%	125%	150%	Mean	75%	100%	125%	150%	Mean	Mean
**Baldo**	80.90	82.77	85.70	88.07	**84.36^{a}**	83.17	84.57	85.98	88.12	**85.46^{b}**	***84.91 A***
**Osmancık**	78.92	81.44	85.77	88.27	**83.60^{c}**	78.70	81.19	86.19	88.06	**83.53^{c}**	***83.57 B***
**Ronaldo**	78.89	82.25	85.70	87.51	**83.58^{c}**	75.00	82.35	86.28	87.83	**82.87^{d}**	***83.22 B***
**Mean**	**79.57^{ns}**	**82.15^{ns}**	**85.72^{ns}**	**87.95^{ns}**	***83.85^{ns}***	**78.96^{ns}**	**82.70^{ns}**	**86.15^{ns}**	**88.00^{ns}**	***83.95^{ns}***	

Resistant Starch (%)

Cultivars/IL	2 Days					4 Days					
	75%	100%	125%	150%	Mean	75%	100%	125%	150%	Mean	Mean
**Baldo**	0.03	0.04	0.38	0.56	**0.25^{ns}**	0.01	0.04	0.52	0.43	**0.25^{ns}**	***0.25 A***
**Osmancık**	0.01	0.02	0.04	0.14	**0.05^{ns}**	0.03	0.06	0.09	0.42	**0.15^{ns}**	***0.10 B***
**Ronaldo**	0.04	0.07	0.31	0.59	**0.25^{ns}**	0.03	0.06	0.24	0.77	**0.28^{ns}**	***0.27 A***
**Mean**	**0.03^{ns}**	**0.05^{ns}**	**0.24^{ns}**	**0.43^{ns}**	***0.19^{ns}***	**0.02^{ns}**	**0.05^{ns}**	**0.28^{ns}**	**0.54^{ns}**	***0.23^{ns}***	

Non-Resistant Starch (%)

Cultivars/IL	2 Days					4 Days					
	75%	100%	125%	150%	Mean	75%	100%	125%	150%	Mean	Mean
**Baldo**	76.73	78.72	82.77	85.54	**80.94^{b}**	77.19	79.27	81.39	84.75	**80.65^{b}**	***80.79 B***
**Osmancık**	71.55	76.49	88.19	89.39	**81.40^{b}**	69.76	79.76	82.79	84.46	**79.19^{c}**	***80.30 B***
**Ronaldo**	78.71	83.43	85.68	87.15	**83.74^{a}**	75.09	83.51	85.86	88.40	**83.22^{a}**	***83.48 A***
**Mean**	**75.66^{f}**	**79.55^{d}**	**85.55^{b}**	**87.36^{a}**	***82.03 A***	**74.01^{g}**	**80.85^{d}**	**83.35^{c}**	**85.87^{b}**	***81.02 B***	

Total Starch (%)

Cultivars/IL	2 Days					4 Days					
	75%	100%	125%	150%	Mean	75%	100%	125%	150%	Mean	Mean
**Baldo**	76.76	78.76	83.15	86.10	**81.19^{b}**	77.20	79.31	81.91	85.18	**80.90^{b}**	***81.05 B***
**Osmancık**	71.56	76.51	88.23	89.53	**81.46^{b}**	69.79	79.82	82.88	84.88	**79.34^{c}**	***80.40 B***
**Ronaldo**	78.75	83.50	85.99	87.74	**84.00^{a}**	75.12	83.58	86.10	89.18	**83.49^{a}**	***83.75 A***
**Mean**	**75.69^{f}**	**79.59^{e}**	**85.79^{b}**	**87.79^{a}**	***82.21 A***	**74.04^{g}**	**80.90^{d}**	**83.63^{c}**	**86.41^{b}**	***81.25 B***	

Crude Protein (%)

Cultivars/IL	2 Days					4 Days					
	75%	100%	125%	150%	Mean	75%	100%	125%	150%	Mean	Mean
**Baldo**	5.57	6.19	6.47	7.05	**6.32^{ab}**	4.94	5.77	6.69	7.72	**6.28^{bc}**	***6.30 A***
**Osmancık**	4.95	5.66	6.13	6.95	**5.92^{d}**	4.98	5.23	6.13	6.55	**5.72^{e}**	***5.82 B***
**Ronaldo**	5.24	5.61	6.30	7.21	**6.09^{cd}**	5.55	6.00	7.15	7.34	**6.51^{a}**	***6.30 A***
**Mean**	**5.25^{e}**	**5.82^{d}**	**6.30^{c}**	**7.07^{a}**	***6.11***	**5.16^{e}**	**5.66^{d}**	**6.66^{b}**	**7.20^{a}**	***6.17***	

Cultivars/IL	Phytic Acid (%)										
	2 Days					4 Days					
	75%	100%	125%	150%	Mean	75%	100%	125%	150%	Mean	Mean
**Baldo**	0.20	0.21	0.22	0.23	**0.22^{c}**	0.14	0.19	0.21	0.25	**0.20^{d}**	***0.21 B***
**Osmancık**	0.17	0.17	0.18	0.22	**0.19^{e}**	0.17	0.18	0.20	0.23	**0.19^{d}**	***0.19 C***
**Ronaldo**	0.28	0.30	0.30	0.31	**0.30^{b}**	0.27	0.33	0.34	0.37	**0.32^{a}**	***0.31 A***
**Mean**	**0.22^{f}**	**0.23^{e}**	**0.24^{d}**	**0.25^{b}**	***0.23 B***	**0.19^{g}**	**0.23^{de}**	**0.25^{c}**	**0.28^{a}**	***0.24 A***	

*NS: Statistically non-significant; different lowercase superscripts indicate interactions, while uppercase superscripts represent the overall means of varieties and irrigation intervals. Values indicated by the same letter are statistically in the same group.

Table S2. The variations in biochemical properties of rice varieties with different ırrigation levels

Amylose (%)				
**Çeşitler/IL**	**75%**	**100%**	**125%**	**150%**
**Baldo**	17.96^{c}	16.33^{d}	14.16^{e}	11.91^{e}
**Osmancık**	21.19^{b}	18.68^{c}	14.02^{e}	11.84^{e}
**Ronaldo**	23.06^{a}	17.70^{c}	14.01^{e}	12.33^{e}
**Mean**	**20.74^{a}**	**17.57^{b}**	**14.07^{c}**	**12.02^{d}**

Amylopectin (%)				
**Çeşitler/IL**	**75%**	**100%**	**125%**	**150%**
**Baldo**	82.04^{d}	83.67^{c}	85.84^{b}	88.09^{a}
**Osmancık**	78.81^{e}	81.32^{d}	85.98^{b}	88.16^{a}
**Ronaldo**	76.94^{f}	82.30^{d}	85.99^{b}	87.67^{a}
**Mean**	**79.26^{d}**	**82.43^{c}**	**85.93^{b}**	**87.98^{a}**

Resistant Starch (%)				
**Çeşitler/IL**	**75%**	**100%**	**125%**	**150%**
**Baldo**	0.02^{d}	0.04^{d}	0.45^{b}	0.49^{b}
**Osmancık**	0.02^{d}	0.04^{d}	0.06^{d}	0.28^{c}
**Ronaldo**	0.03^{d}	0.07^{d}	0.28^{c}	0.68^{a}
**Mean**	**0.03^{c}**	**0.05^{c}**	**0.26^{b}**	**0.49^{a}**

Non-Resistant Starch (%)				
**Çeşitler/IL**	**75%**	**100%**	**125%**	**150%**
**Baldo**	76.96^{f}	79.00^{e}	82.08^{d}	85.14^{b}
**Osmancık**	70.66^{g}	78.12^{e}	85.49^{b}	86.92^{a}
**Ronaldo**	76.90^{f}	83.47^{c}	85.77^{b}	87.78^{a}
**Mean**	**74.84^{d}**	**80.20^{c}**	**84.45^{b}**	**86.61^{a}**

Total Starch (%)				
**Çeşitler/IL**	**75%**	**100%**	**125%**	**150%**
**Baldo**	76.98^{f}	79.04^{e}	82.53^{d}	85.64^{c}
**Osmancık**	70.67^{g}	78.17^{e}	85.55^{c}	87.20^{b}
**Ronaldo**	76.93^{f}	83.54^{d}	86.05^{c}	88.46^{a}
**Mean**	**74.86^{d}**	**80.25^{c}**	**84.71^{b}**	**87.10^{a}**

Crude Protein (%)				
**Çeşitler/IL**	**75%**	**100%**	**125%**	**150%**
**Baldo**	5.25^{ef}	5.98^{c}	6.58^{b}	7.39^{a}
**Osmancık**	4.97^{f}	5.44^{de}	6.13^{c}	6.75^{b}
**Ronaldo**	5.40^{e}	5.80^{cd}	6.72^{b}	7.28^{a}
**Mean**	**5.21^{c}**	**5.74^{c}**	**6.48^{b}**	**7.14^{a}**

Phtic Acid (%)				
**Çeşitler/IL**	**75%**	**100%**	**125%**	**150%**
**Baldo**	0.17^{g}	0.20^{f}	0.21^{e}	0.24^{d}
**Osmancık**	0.17^{g}	0.18^{g}	0.19^{f}	0.22^{e}
**Ronaldo**	0.27^{c}	0.31^{b}	0.32^{b}	0.34^{a}
**Mean**	**0.21^{d}**	**0.23^{c}**	**0.24^{b}**	**0.27^{a}**

* Values indicated by the same letter are statistically in the same group.

Table S3. The effects of different ırrigation levels and ıntervals on the mineral content of rice varieties

B (ppm)											
Cultivars/IL	2 Days					4 Days					
	75%	100%	125%	150%	Mean	75%	100%	125%	150%	Mean	Mean
Baldo	7.22	8.00	13.54	16.06	11.20^{d}	9.73	15.24	13.45	25.81	16.06^{a}	13.63 B
Osmancık	7.26	9.21	10.46	12.67	9.90^{e}	10.04	13.78	14.02	15.52	13.34^{c}	11.62 C
Ronaldo	13.16	15.01	16.96	17.69	15.71^{ab}	8.49	11.22	14.36	23.79	14.47^{bc}	15.09 A
Mean	9.21^{d}	10.74^{d}	13.66^{c}	15.47^{b}	12.27 B	9.42^{d}	13.42^{c}	13.94^{bc}	21.71^{a}	14.62 A	

Ca (ppm)											
Cultivars/IL	2 Days					4 Days					
	75%	100%	125%	150%	Mean	75%	100%	125%	150%	Mean	Mean
Baldo	231.23	270.25	256.57	435.52	298.39^{d}	279.97	363.01	373.61	438.61	363.80^{a}	331.10 A
Osmancık	218.50	239.77	331.27	420.17	302.42^{cd}	248.26	311.41	334.62	350.21	311.12^{c}	306.77 B
Ronaldo	219.88	303.36	378.66	397.25	324.79^{b}	220.86	269.12	320.58	382.12	298.17^{d}	311.48 B
Mean	223.20^{g}	271.12^{e}	322.17^{d}	417.65^{a}	308.54 B	249.70^{f}	314.51^{d}	342.94^{c}	390.32^{b}	324.37 A	

Cu (ppm)											
Cultivars/IL	2 Days					4 Days					
	75%	100%	125%	150%	Mean	75%	100%	125%	150%	Mean	Mean
Baldo	150.56	169.53	166.79	174.38	165.32^{b}	164.81	210.53	205.58	241.22	205.54^{a}	185.43 A
Osmancık	139.31	162.74	154.86	175.37	158.07^{c}	135.30	159.11	173.20	181.72	162.33^{bc}	160.20 B
Ronaldo	143.07	144.79	189.45	193.64	167.74^{b}	137.69	118.27	182.75	184.38	155.77^{c}	161.76 B
Mean	144.31^{e}	159.02^{d}	170.37^{c}	181.13^{b}	163.71 B	145.94^{e}	162.64^{d}	187.18^{b}	202.44^{a}	174.55 A	

Fe (ppm)											
Cultivars/IL	2 Days					4 Days					
	75%	100%	125%	150%	Mean	75%	100%	125%	150%	Mean	Mean
Baldo	67.83	76.98	87.98	85.50	79.57^{ns}	79.32	97.81	104.52	122.05	100.92^{ns}	90.25 A
Osmancık	46.70	64.42	81.17	82.24	68.63^{ns}	74.53	83.38	86.74	100.95	86.40^{ns}	77.52 B
Ronaldo	57.08	58.43	93.76	99.96	77.31^{ns}	72.16	76.26	131.73	112.32	98.12^{ns}	87.71 A
Mean	57.20^{ns}	66.61^{ns}	87.64^{ns}	89.23^{ns}	75.17 B	75.34^{ns}	85.82^{ns}	107.66^{ns}	111.77^{ns}	95.15 A	

K (ppm)											
Cultivars/IL	2 Days					4 Days					
	75%	100%	125%	150%	Mean	75%	100%	125%	150%	Mean	Mean
Baldo	564.29	620.83	644.80	723.78	638.42^{d}	653.41	962.51	995.15	1287.59	974.67^{a}	806.54 A
Osmancık	714.48	791.87	910.43	1031.29	862.02^{b}	748.02	767.85	818.57	596.53	732.75^{c}	797.38 A
Ronaldo	581.02	606.20	863.02	845.70	723.99^{c}	490.47	545.12	686.78	798.76	630.28^{d}	677.13 B
Mean	619.93^{ns}	672.96^{ns}	806.08^{ns}	866.92^{ns}	741.48 B	630.63^{ns}	758.49	833.50	894.30	779.23 A	

Mg (ppm)											
Cultivars/IL	2 Days					4 Days					
	75%	100%	125%	150%	Mean	75%	100%	125%	150%	Mean	Mean
Baldo	221.39	238.10	271.80	321.18	263.12^{e}	217.56	435.19	494.53	309.16	364.11^{b}	313.61 C
Osmancık	221.41	264.25	278.99	306.61	267.82^{e}	252.53	299.23	281.02	339.92	293.18^{d}	280.50 B
Ronaldo	313.82	328.07	400.67	464.75	376.83^{a}	229.20	287.61	366.31	475.01	339.53^{c}	358.18 A
Mean	252.21^{f}	276.81^{e}	317.16^{d}	364.18^{b}	302.59 B	233.10^{g}	340.68^{c}	380.62^{a}	374.70^{ab}	332.27 A	

					**Mn (ppm)**						
**Cultivars/IL**	**2 Days**					**4 Days**					
	**75%**	**100%**	**125%**	**150%**	**Mean**	**75%**	**100%**	**125%**	**150%**	**Mean**	**Mean**
**Baldo**	3.01	3.14	3.48	3.19	**3.21**^{e}	2.98	6.42	6.54	7.98	**5.98**^{c}	***4.59 B***
**Osmancık**	3.65	3.79	4.79	4.38	**4.15**^{d}	5.03	5.28	5.85	6.27	**5.61**^{c}	***4.88 B***
**Ronaldo**	6.80	7.54	8.50	10.77^{b}	**8.40**^{b}	8.38	9.49	11.35	14.98	**11.05**^{a}	***9.73 A***
**Mean**	**4.49**^{f}	**4.82**^{f}	**5.59**^{de}	**6.12**^{d}	***5.25 B***	**5.47**^{e}	**7.06**^{c}	**7.91**^{b}	**9.74**^{a}	***7.55 A***	
					**Na (ppm)**						
**Cultivars/IL**	**2 Days**					**4 Days**					
	**75%**	**100%**	**125%**	**150%**	**Mean**	**75%**	**100%**	**125%**	**150%**	**Mean**	**Mean**
**Baldo**	324.05	365.38	342.56	439.10	**367.77**^{d}	412.85	505.24	520.43	544.12	**495.66**^{a}	***431.72 A***
**Osmancık**	331.71	373.69	378.66	424.51	**377.14**^{cd}	338.34	356.25	388.13	403.30	**371.50**^{d}	***374.32 C***
**Ronaldo**	315.66	347.99	428.80	442.81	**383.81**^{c}	319.02	386.56	450.11	457.17	**403.21**^{b}	***393.51 B***
**Mean**	**323.80**^{g}	**362.36**^{f}	**383.34**^{e}	**435.47**^{c}	***376.24 B***	**356.74**^{f}	**416.02**^{d}	**452.89**^{b}	**468.20**^{a}	***423.46 A***	
					**P (ppm)**						
**Cultivars/IL**	**2 Days**					**4 Days**					
	**75%**	**100%**	**125%**	**150%**	**Mean**	**75%**	**100%**	**125%**	**150%**	**Mean**	**Mean**
**Baldo**	935.89	1007.82	1108.97	1265.16	**1079.46**^{d}	928.89	1454.15	1696.52	1940.20	**1504.94**^{a}	***1292.20 B***
**Osmancık**	983.35	934.44	1056.93	1218.80	**1048.38**^{d}	1076.81	1319.38	1197.52	1016.02	**1152.43**^{c}	***1100.41 C***
**Ronaldo**	1349.12	1370.71	1646.10	1722.77	**1522.17**^{a}	933.55	1173.43	1496.79	1601.87	**1301.41**^{b}	***1411.79 A***
**Mean**	**1089.45**^{ef}	**1104.32**^{e}	**1270.66**^{d}	**1402.24**^{bc}	***1216.67 B***	**979.75**^{f}	**1315.65**^{cd}	**1463.61**^{ab}	**1519.36**^{a}	***1319.59 A***	
					**S (ppm)**						
**Cultivars/IL**	**2 Days**					**4 Days**					
	**75%**	**100%**	**125%**	**150%**	**Mean**	**75%**	**100%**	**125%**	**150%**	**Mean**	**Mean**
**Baldo**	1058.47	1078.26	1318.22	1250.76	**1176.43**^{c}	1143.62	1446.37	2340.47	1621.01	**1637.86**^{a}	***1407.15 B***
**Osmancık**	1031.29	1057.81	1119.05	1273.56	**1120.43**^{c}	1196.40	1536.24	1707.83	1498.78	**1484.81**^{b}	***1302.62 C***
**Ronaldo**	1420.15	1528.27	1801.62	1912.66	**1665.67**^{a}	1099.64	1368.17	1588.05	1562.30	**1404.54**^{b}	***1535.11 A***
**Mean**	**1169.97**^{de}	**1221.45**^{d}	**1412.96**^{c}	**1478.99**^{c}	***1320.84 B***	**1146.56**^{e}	**1450.26**^{c}	**1878.78**^{a}	**1560.69**^{b}	***1509.07 A***	
					**Zn (ppm)**						
**Cultivars/IL**	**2 Days**					**4 Days**					
	**75%**	**100%**	**125%**	**150%**	**Mean**	**75%**	**100%**	**125%**	**150%**	**Mean**	**Mean**
**Baldo**	42.33	52.20	54.75	59.51	**52.20**^{bc}	53.15	67.38	71.03	81.34	**68.23**^{a}	***60.21 A***
**Osmancık**	41.79	51.28	58.55	56.05	**51.92**^{c}	48.63	44.59	49.50	53.00	**48.93**^{d}	***50.42 C***
**Ronaldo**	42.75	42.99	57.11	64.93	**51.95**^{c}	42.36	51.83	58.89	62.99	**54.02**^{b}	***52.98 B***
**Mean**	**42.29**^{e}	**48.82**^{d}	**56.80**	**60.16**^{b}	***52.02 B***	**48.05**^{d}	**54.60**^{c}	**59.80**^{c}	**65.78**^{a}	***57.06 A***	

*NS: Statistically non-significant; different lowercase superscripts indicate interactions, while uppercase superscripts represent the overall means of varieties and irrigation intervals. Values indicated by the same letter are statistically in the same group.

Table S4. The variations in mineral content of rice varieties with different ırrigation levels

		B (ppm)					Ca (ppm)		
**Çeşitler/IL**	**75%**	**100%**	**125%**	**150%**	**Çeşitler/IL**	**75%**	**100%**	**125%**	**150%**
**Baldo**	8.47^{g}	11.62^{ef}	13.49^{c}	20.94^{a}	**Baldo**	255.60^{g}	316.63^{de}	315.09^{e}	437.07^{a}
**Osmancık**	8.65^{g}	11.50^{ef}	12.24^{de}	14.09^{c}	**Osmancık**	233.38^{h}	275.59^{f}	332.95^{d}	385.19^{b}
**Ronaldo**	10.82^{f}	13.11^{cd}	15.66^{b}	20.74^{a}	**Ronaldo**	220.37^{h}	286.24^{f}	349.62^{c}	389.69^{b}
**Mean**	**9.32^{d}**	**12.08^{c}**	**13.80^{b}**	**18.59^{a}**	**Mean**	**236.45^{d}**	**292.82^{c}**	**332.55^{b}**	**403.98^{a}**

		Cu (ppm)					Fe (ppm)		
**Çeşitler/IL**	**75%**	**100%**	**125%**	**150%**	**Çeşitler/IL**	**75%**	**100%**	**125%**	**150%**
**Baldo**	157.69^{d}	190.03^{b}	186.19^{bc}	207.80^{a}	**Baldo**	73.57^{f}	87.39^{de}	96.25^{c}	103.77^{b}
**Osmancık**	137.30^{e}	160.92^{d}	164.03^{d}	178.55^{c}	**Osmancık**	60.62^{h}	73.90^{f}	83.95^{e}	91.60^{cd}
**Ronaldo**	140.38^{e}	131.53^{e}	186.10^{bc}	189.01^{b}	**Ronaldo**	64.62^{gh}	67.35^{g}	112.74^{a}	106.14^{b}
**Mean**	**145.13^{d}**	**160.83^{c}**	**178.77^{b}**	**191.79^{a}**	**Mean**	**66.27^{c}**	**76.21^{b}**	**97.65^{a}**	**100.50^{a}**

		K (ppm)					Mg (ppm)		
**Çeşitler/IL**	**75%**	**100%**	**125%**	**150%**	**Çeşitler/IL**	**75%**	**100%**	**125%**	**150%**
**Baldo**	608.85^{g}	791.67^{cde}	819.98^{cd}	1005.69^{a}	**Baldo**	219.48^{h}	336.65^{c}	383.16^{b}	315.17^{de}
**Osmancık**	731.25^{f}	779.86^{de}	864.50^{b}	813.91^{cde}	**Osmancık**	236.97^{g}	281.74^{f}	280.01^{f}	323.27^{cd}
**Ronaldo**	535.74^{h}	575.66^{gh}	774.90^{e}	822.23^{c}	**Ronaldo**	271.51^{f}	307.84^{e}	383.49^{b}	469.88^{a}
**Mean**	**625.28^{d}**	**715.73^{c}**	**819.79^{b}**	**880.61^{a}**	**Mean**	**242.65^{d}**	**308.74^{c}**	**348.89^{b}**	**369.44^{a}**

		Mn (ppm)					Na (ppm)		
**Çeşitler/IL**	**75%**	**100%**	**125%**	**150%**	**Çeşitler/IL**	**75%**	**100%**	**125%**	**150%**
**Baldo**	3.00^{h}	4.78^{fg}	5.01^{efg}	5.59^{e}	**Baldo**	368.45^{f}	435.31^{c}	431.49^{c}	491.61^{a}
**Osmancık**	4.34g	4.54g	5.32^{ef}	5.33^{ef}	**Osmancık**	335.02^{g}	364.97^{f}	383.39^{e}	413.91^{d}
**Ronaldo**	7.59^{d}	8.51^{c}	9.93^{b}	12.87^{a}	**Ronaldo**	317.34^{h}	367.27^{f}	439.45^{bc}	449.99^{b}
**Mean**	**4.98^{d}**	**5.94^{c}**	**6.75^{b}**	**7.93^{a}**	**Mean**	**340.27^{d}**	**389.19^{c}**	**418.11^{b}**	**451.83^{a}**

		P (ppm)					S (ppm)		
**Çeşitler/IL**	**75%**	**100%**	**125%**	**150%**	**Çeşitler/IL**	**75%**	**100%**	**125%**	**150%**
**Baldo**	932.39^{g}	1230.99^{cd}	1402.74^{b}	1602.68^{a}	**Baldo**	1101.04^{f}	1262.31^{e}	1829.34^{a}	1435.88^{c}
**Osmancık**	1030.08^{fg}	1126.91^{ef}	1127.23^{ef}	1117.41^{ef}	**Osmancık**	1113.85^{f}	1297.03^{de}	1413.44^{c}	1386.17^{cd}
**Ronaldo**	1141.33^{de}	1272.07^{c}	1571.44^{a}	1662.32^{a}	**Ronaldo**	1259.90^{e}	1448.22^{c}	1694.83^{b}	1737.48^{ab}
**Mean**	**1034.60^{d}**	**1209.99^{c}**	**1367.14^{b}**	**1460.80^{a}**	**Mean**	**1158.26^{d}**	**1335.85^{c}**	**1645.87^{a}**	**1519.84^{b}**

		Zn (ppm)		
**Çeşitler/IL**	**75%**	**100%**	**125%**	**150%**
**Baldo**	47.74^{e}	59.79^{c}	62.89^{b}	70.43^{a}
**Osmancık**	45.21^{ef}	47.94^{e}	54.02^{d}	54.52^{d}
**Ronaldo**	42.56^{f}	47.41^{e}	58.00^{c}	63.96^{b}
**Mean**	**45.17^{d}**	**51.71^{c}**	**58.30^{b}**	**62.97^{a}**

*Values indicated by the same letter are statistically in the same group.
